# ‘Return to Work’ Coordinator Model and Work Participation of Employees: A Natural Intervention Study in Finland

**DOI:** 10.1007/s10926-021-09970-x

**Published:** 2021-04-07

**Authors:** Johanna Kausto, Tuula Oksanen, Aki Koskinen, Jaana Pentti, Pauliina Mattila-Holappa, Leena Kaila-Kangas, Nina Nevala, Mika Kivimäki, Jussi Vahtera, Jenni Ervasti

**Affiliations:** 1 Finnish Institute of Occupational Health, Topeliuksenkatu 40, 00250 Helsinki, Finland; 2 School of Medicine, Institute of Public Health and Clinical Nutrition, University of Eastern Finland, Kuopio, Finland; 3 Department of Public Health, University of Turku, and Centre for Population Health Research, University of Turku and Turku University Hospital, Turku, Finland; 4 Clinicum, Faculty of Medicine, University of Helsinki, Helsinki, Finland; 5 Department of Epidemiology and Public Health, University College London, London, UK

**Keywords:** Return to work coordination, Work ability, Municipalities, Sickness absence, Disability management

## Abstract

*Purpose* Employers increasingly use ‘return to work’ (RTW) coordinators to support work ability and extend working careers, particularly among employees with reduced work ability. We examined whether applying this model was associated with changes in employee sickness absence and disability retirements. *Methods* We used data from the Finnish Public Sector study from 2009 until 2015. Employees where the model was introduced in 2012 constituted the cases (n = 4120, one municipality) and employees where the model was not in use during the follow-up, represented the controls (n = 5600, two municipalities). We analysed risk of disability retirement in 2013–2015 and risk of sickness absence after (2013–2015) vs. before (2009–2011) intervention by case–control status. *Results* The incidence of disability retirement after the intervention was lower in cases compared to controls both in the total population (hazard ratio HR = 0.49, 95% CI 0.30–0.79) and in the subgroup of participants with reduced work ability (HR = 0.34, 95% CI 0.12–0.99). The risk of sickness absence increased from pre-intervention to post-intervention period both among cases and controls although the relative increase was greater among cases (RR_post- vs. pre-intervention_ = 1.26, 95% CI 1.14–1.40) than controls (RR_post- vs. pre-intervention_ = 1.03, 95% CI 0.97–1.08). In the group of employees with reduced work ability, no difference in sickness absence trends between cases and controls was observed. *Conclusions* These findings suggest that RTW-coordinator model may increase employee sickness absence, but decrease the risk of disability retirement, i.e., permanent exclusion from the labour market.

## Introduction

As populations are ageing, extending working careers is a common goal globally. Vocational rehabilitation serves this end and better integration and coordination of services and procedures in vocational rehabilitation have been sought in many countries [1, 2]. Return to work (RTW) coordinators have been regarded as important actors in this process [3–6]. The main goal of RTW-coordinators is to increase the return to work after work disability and work participation of employees with sickness absence history and reduced work ability.

A recent study on the practices of RTW-coordinators concluded that despite a wide variety of contexts and diverging definitions of competencies needed, a set of common RTW-coordination practices appears to exist across countries [4]. These include applying laws, policies, and regulations related to social insurance and RTW, contacting the worker, and planning the individually tailored return to work process. The clients of RTW-coordinators are employees who have health problems or permanent disabilities, who struggle to maintain their work ability, and are at risk of long-term work disability. In most cases, RTW-coordinators also assist managers and the whole organization in supporting work ability and well-being at work. Co-operation across different jurisdictions is an essential element of the job. In Finland, organizational procedures and practices that aim to support work ability and extend working careers are commonly used at workplaces, although the work task of return to work- coordinator is rather new.

To date, evidence on the effectiveness of RTW-coordinator model is mixed and inconclusive. There is some research suggesting that the RTW-coordinator model or some components of the model might decrease work disability duration [7, 8], and other studies arguing that offering RTW-coordination had no benefits when compared to usual practice [9, 10]. The aim of this study was to examine whether applying RTW-coordinator practice in the Finnish public sector affected the probability of work participation of employees, especially those with reduced work ability.

## Methods

### Study Design and Population

Participants were from the Finnish Public Sector (FPS) study cohort [11], which represents about 26% of Finnish public sector workers. This large follow-up study links responses from a questionnaire survey on work, lifestyle, and health in 2012 to individual level records from several national and employer registers. In 2019, we additionally interviewed the representatives of seven of the FPS-municipalities on their work disability prevention practices, including RTW coordinator model.

With one exception, all municipalities had implemented RTW-coordinator model at some point between 2007 and 2017. To achieve the desired design with sufficient follow-up both before and after the intervention and control group without intervention during the follow-up period, we were left with four municipalities (two case and two control municipalities) to be included in our sample. The interventions were carried out in the case municipalities in 2011 and 2012. Two municipalities implemented their RTW-coordinator models after the follow-up ended in 31.12.2015, and they constituted the control group. To meet the parallel trends-assumption of difference-in-differences analysis, we examined the trends in sickness absence before the intervention. One of the case municipalities had to be omitted due not meeting the parallel trends assumption (the control group differed statistically significantly from the case group; p < 0.001 for preintervention time × status interaction term in the total sample and p = 0.006 in those with reduced work ability). Thus, our final analytic sample consisted of employees of one case municipality and two control municipalities (p = 0.06 for preintervention time × status interaction term in the total sample and p = 0.75 in those with reduced work ability). We included participants who were aged 18 to 65 at the time of the intervention and were not on a long-term (more than 90 days) sickness absence or were not granted disability pension in two years before the intervention (n = 9720). 90 days is the threshold for a long-term sickness absence in the Finnish sickness benefit system. Pre-intervention follow-up was from Jan 1, 2009 to Dec 31, 2011 and post-intervention follow-up was from Jan 1, 2013 to Dec 31, 2015. The ethics committee of the Hospital District of Helsinki and Uusimaa has approved the FPS study, and the ethics committee of the Finnish Institute of Occupational Health has approved the sub-study including the interviews.

### Study Context

Public sector is a significant employer and branch of industry in Finland engaging currently nearly half a million of all 5.5 million employees in this country. In Finland, all non-retired residents aged 16 to 67 are eligible for a compensation of absence from work due to own illness. When employment has lasted for at least one month, in case of work disability due to illness, the employer continues paying salary on the day on which the illness begins plus the following nine weekdays. According to collective labour unions’ negotiated agreements, many employers continue paying full salary for the first months. After this, the Social Insurance Institution of Finland starts paying statutory sickness benefit, which compensates progressively for lost wage income. Employers are obligated to inform occupational health care when an employee has been absent from work for 30 calendar days [12]. When sick leave has lasted for 60 days, the employer must apply for sickness benefit from the Social Insurance Institution of Finland and the occupational health care must evaluate the rehabilitation needs of the employee. When sickness benefit has been paid for a total of 90 days, occupational health care evaluates the work ability, and negotiates about the options of return to work with the employee and the employer. The maximum length of sickness absence compensation is 300 working days per disease in two years. In case of long-term work disability, a disability pension can be granted either temporarily or permanently.

### Intervention: The RTW-Coordinator Model

RTW-coordinators are employed by different types of Finnish public and private organizations. In municipalities, they are usually a part of Human Resources (HR) organization. The focus of their work is on planning and follow-up of organizational practices and processes supporting work ability, workplace assessment and finding solutions to support employees with reduced work ability planning workplace accommodation (i.e. changes in work schedules, work organization, work environment, assistive technology, and assistance). Coordination of different services and co-operation between employer, occupational health care, rehabilitation, and insurance companies is important [8]. Knowledge on labour market, social insurance, and legal aspects of work disability, and skills on communication and problem-solving are required in the task [13]. Implementing a RTW -coordinator model is not mandatory but recommended in Finland. There are no official qualifications for the job, but a RTW work coordinator is usually a professional of working life, rehabilitation, the service system, and client work. The number of RTW coordinators was increased after a training project carried out as a part of a national project in 2015–2018 [14].

### Case–Control-Status

If the employer (municipality) had implemented RTW-coordinator model (City B), the employees were regarded in the analyses as cases (= 1). If the employer had not implemented such model, the employees were regarded as controls (= 0).

### Measures of Reduced work Ability

Chronic somatic illnesses were derived from the National register of special reimbursement for medication (valid at the beginning of the follow-up). They included type 1 and type 2 diabetes, chronic heart diseases, rheumatoid arthritis, chronic asthma and COPD, stage 2 hypertension, Parkinson’s disease, epilepsy, uremia, bowel disease, MS disease, and diseases of the pancreas. Information on mental illnesses was derived from survey data (self-reported doctor-diagnosed diseases) and included depression and other mental disorders. We coded these as: at least one chronic disease or illness (= 1), and no chronic disease or illness (= 0).

Self-rated work ability was assessed using the first dimension of the Work ability Index (WAI), in which the respondent estimates with a ten-point scale his or her current work ability compared to the lifetime best (0 = not able to work–10 = lifetime best). Previous studies have shown this single item question to be highly associated with the overall WAI [15, 16]. Reduced work ability was defined in this study as having at least one chronic disease or illness and self-rated less than good (0–6) work ability. Employees with reduced work ability (n = 683) contributed to the subsample of the study.

### Sickness Absence and Disability Retirement

We calculated the annual total number of sickness absence days (person-year weighted mean) derived from the employers’ registers in 1.1.2009–31.12.2011 (pre-intervention time) and 1.1.2013– 31.12.2015 (post-intervention time). That is, all employees were included in the analyses, not only those with sickness absence. The date of granting full-time permanent disability pension during 1.1.2013–31.12.2015 was derived from the Finnish Centre for Pensions.

### Covariates

Information on age, sex, job contract (permanent or temporary), socioeconomic status (SES), and previous long-term sickness absence (of > 30 days) was obtained from the employers’ registers. The occupations were classified according to the 2001 International Standard Classification of Occupations codes (ISCO) and we used the 1-digit level for categorising them into three levels: high (upper-grade nonmanual worker including managers, administrators, and specialists), intermediate (lower-grade nonmanual workers including office workers, clerks, customer service and sales workers, and hospital nurses), and low (manual workers including construction workers, manufacturing and transportation workers). These classes generally distinguish between different types of jobs and work exposures, e.g. between exposure to heavy physical work and sedentary work.

Information on health behaviours and mental health was derived from the survey [11]. Smoking was coded as a binary variable (“current smoker vs. not current smoker”). Alcohol use was assessed by participants’ weekly consumption of alcohol. One drink was approximately equivalent to one unit or one glass of alcoholic drink or 12 g of alcohol. Alcohol intake was dichotomized into no use, moderate use (a maximum of 140 g, equaling 12 units for women; and 280 g, equaling 23 units for men), and heavy alcohol use (more than 12/23 units per week) [17]. Leisure-time physical inactivity was assessed using a question requesting weekly time spent for physical activity at the moderate-to-heavy level. Participants were categorized as being physically inactive if they reported less than two metabolic equivalent task hours per day (approximately 30 min. of walking) and active if more than this [18]. The General Health Questionnaire (GHQ-12) was used to evaluate psychiatric distress [19]. In GHQ-12, respondents rated how much they were affected by each of the 12 symptoms of distress (0 = not at all, 0 = the same as usual, 1 = slightly more than usual, 1 = much more than usual). Participants with a rating of 1 in at least 4 items of the total measure were coded as cases of psychiatric distress.

### Statistical Analyses

We applied a quasi-experimental study design with difference-in-differences (DID) method. This analysis is anticipated to control for fixed unobserved individual-level confounders and common trends affecting case and control groups [20–22]. An assumption in the DID-analysis is that the trends of the outcomes are parallel in case and control groups before the intervention. To examine the risk of sickness absence during the 3 years after relative to 3 years before RTW-coordinator model, we applied repeated-measures negative binomial regression analysis using the GEE method with exchangeable correlation structure (considering the intraindividual correlation between measurements). Cox proportional hazards regression analysis was applied to examine risk of disability pension after implementation of RTW-coordinator model.

Trends in the annual total number of sickness absence days were followed from year 2009 to year 2011 (pre-intervention) and from year 2013 to year 2015 (post-intervention). Regarding analysis on disability retirement the follow-up was from Jan 1, 2013 until full time work disability onset, death, or end of the follow-up (Dec 31, 2015), whichever occurred first. We conducted analyses both in the total population and in those with reduced work ability. Post vs. pre-intervention risk ratio (RR with 95% confidence intervals, CI) for sickness absence and hazard ratio (HR with 95% CI) for disability retirement for ‘case’ status with ‘control’ status as reference were adjusted for sex, age, SES, and lifestyle factors of smoking, alcohol use, and leisure-time physical inactivity. In analysis of disability pension, additional adjustment was made for long-term sickness absence history (> 30 days sickness absence episodes during 2 years before the follow-up for disability pension). To analyze whether the trend of sickness absence differed post- vs.- pre-intervention among cases and controls (DID-analysis), we tested time × status interaction. SAS software package (version 9.4; SAS Institute, Inc, Cary, North Carolina) was used for statistical analyses.

## Results

In the total study population, the proportion of women was higher (83%) among cases (RTW-coordinator model implemented) than controls (no RTW-coordinator model in use) (78%). Among those with reduced work ability, no corresponding difference was found. Of the occupational classes, upper-grade non manual work was the most prevalent (60% among cases and controls in the total population and 65% among cases and 57% among controls in the reduced work ability group). Manual workers, temporary job contracts and previous long-term sickness absence (of > 30 days) were more prevalent among cases than controls (Table 1).

**Table 1:** Descriptive statistics of the study population by case/control group status, n (%)

	Total population (n = 9720)				Employees with reduced work ability (n = 683)			
	Cases (n = 4120)	Controls (n = 5600)		p for difference	Cases (n = 281)		Controls (n = 402)	p for difference
Sex				< 0.001				0.43
Men	697 (17)	1243 (22)			70 (25)	111 (28)		
Women	3423 (83)	4357 (78)			211 (75)	291 (72)		
Mean age (SD)	47.7 (8.9)	48.1 (8.7)		0.01	49.9 (8.2)	50.8 (7.3)		0.14
Occupational class				< 0.001				0.001
High	2403 (60)	3221 (60)			176 (65)	223 (57)		
Intermediate	344 (9)	1031 (19)			42 (16)	108 (28)		
Low	1212 (31)	1161 (21)			50 (19)	59 (15)		
Job contract				< 0.001				< 0.001
Permanent	3668 (89)	5526 (99)			261 (93)	399 (99)		
Temporary	452 (11)	66 (1)			20 (7)	3 (1)		
Smoking				0.22				0.12
Yes	550 (14)	799 (14)			55 (20)	98 (25)		
Drinking				0.34				0.98
No	693 (17)	971 (17)			57 (20)	82 (20)		
Moderate	3028 (74)	4133 (74)			187 (67)	265 (66)		
Heavy	399 (9)	496 (9)			37 (13)	55 (14)		
Physically inactive								
Yes	1009 (25)	1532 (27)		0.001	119 (42)	188 (47)		0.23
Psychiatric distress								
Yes	947 (23)		1234 (22)	0.27	167 (59)	227 (57)		0.46
Sickness absence of > 30 days (in 2 years pre-intervention)								
Yes	370 (9)	89 (2)		< 0.001	59 (21)	13 (3)		< 0.001
Pooled mean number* (SD) of sickness absence days								
Three years before	12.5 (14.4)	16.3 (21.3)			23.6 (20.3)	30.0 (32.9)		
Three years after	13.8 (20.4)	16.0 (22.0)			24.8 (29.4)	29.3 (32.3)		

*Pooled mean = (Total number of sickness absence days _year 1_ + total number of sickness absence days _year 2_ + total number of sickness absence days _year 3_)/3

**Fig. 1:**
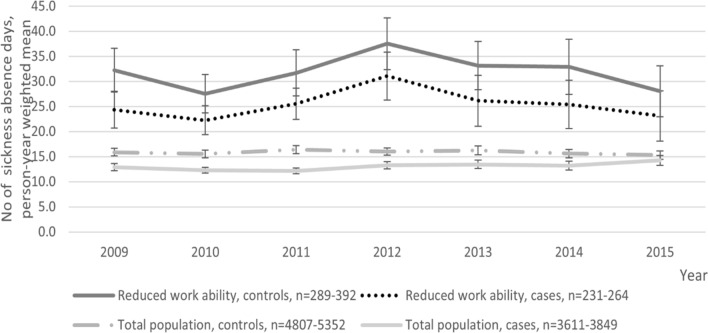
Trends in annual mean numbers of sickness absence days (person-year weighted mean, 95% CI) from pre-implementation (years 2009–2011) to implementation and wash-out (year 2012), and post-implementation (years 2013–2015) period

The risk of sickness absence increased from pre-intervention period to post-intervention period (three years after implementing the RTW-coordinator model as compared to three years preceding the intervention) both among cases and controls. After adjustment for sex, age, SES, job contract, psychiatric distress, and health behaviour the increase in the risk was 1.26-fold (95% CI 1.14–1.40) among cases and 1.03-fold (95% CI 0.97–1.08) among controls in the total population. The trends in sickness absence differed post-vs. pre-intervention statistically significantly (p < 0.001) between cases and controls in the total population. In those with reduced work ability, the increase in the risk of sickness absence after the intervention was 1.80-fold (95% CI 1.17–2.78) among cases and 1.27-fold (95% CI 1.02–1.59) among controls, respectively. The trends were not statistically different from each other among employees with reduced work ability (Table 2 and Fig. 1).

**Table 2:** Ratio of annual days of sickness absence after versus before implementing RTW-coordinator model by case/control group status. Generalized estimating equations analysis

	Cases		Controls		Group x time interaction, p-value
	RR_post- vs. pre-intervention_	95% CI	RR_post- vs. pre-intervention_	95% CI	
Model 1					
Sickness absence days, Total population					
Three years before	1		1		
Three years after	1.29	1.16–1.44	1.04	0.98–1.07	0.001
Sickness absence days, Reduced work ability					
Three years before	1		1		
Three years after	1.80	1.17–2.75	1.34	1.07–1.68	0.82
Model 2					
Sickness absence days, Total population					
Three years before	1		1		
Three years after	1.26	1.14–1.40	1.03	0.97–1.08	0.0012
Sickness absence days, Reduced work ability					
Three years before	1		1		
Three years after	1.80	1.17–2.78	1.27	1.02–1.59	0.83

Model 1: Adjusted for sex, age, SES, and job contract

Model 2: Adjusted as Model 1 + psychiatric distress, health behaviour (smoking, alcohol intake and leisure-time physical inactivity)

**Fig. 2:**
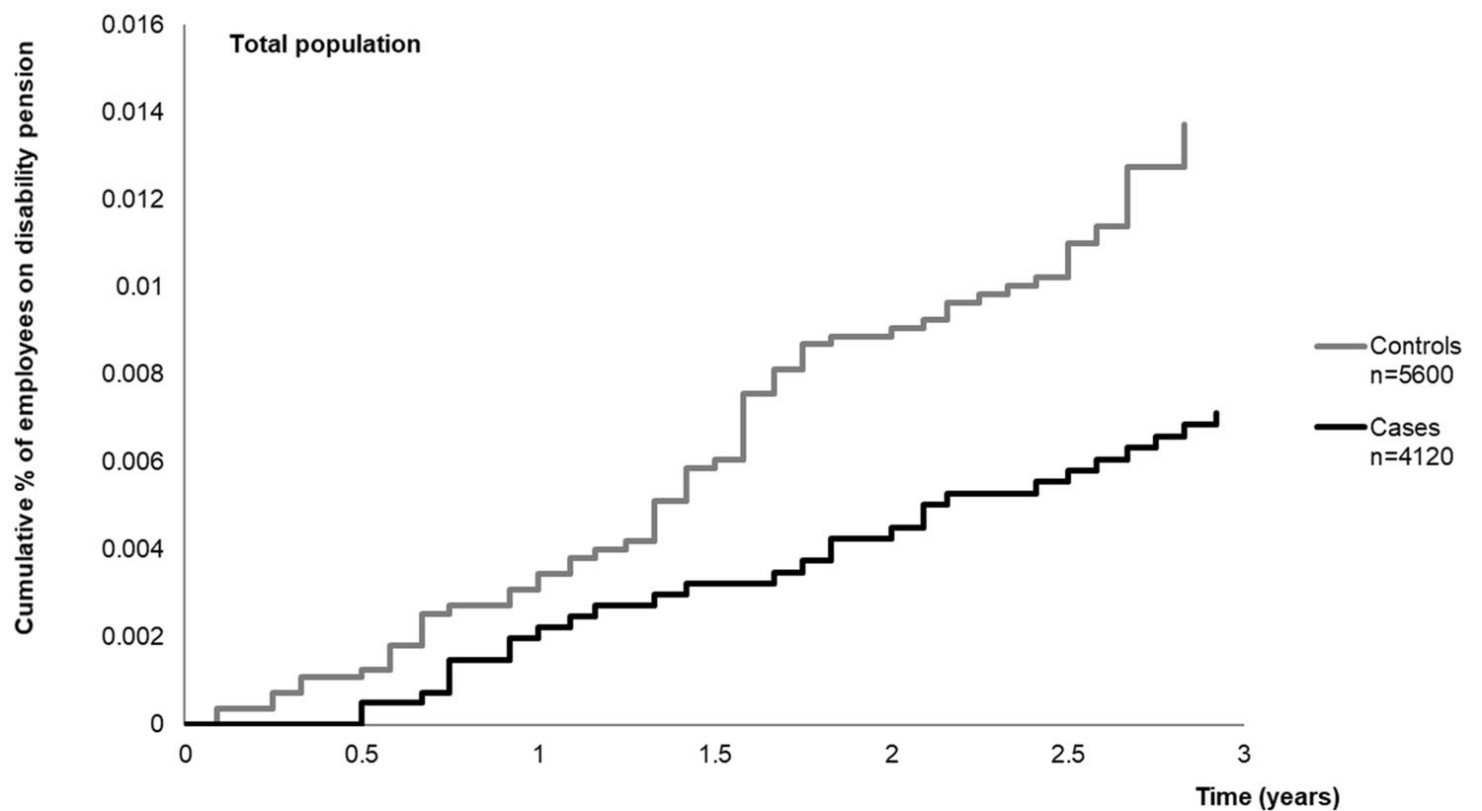
Cumulative incidence of disability retirement after implementation of RTW-coordinator model stratified by case/control status in total population (Kaplan–Meier hazard functions)

**Fig. 3:**
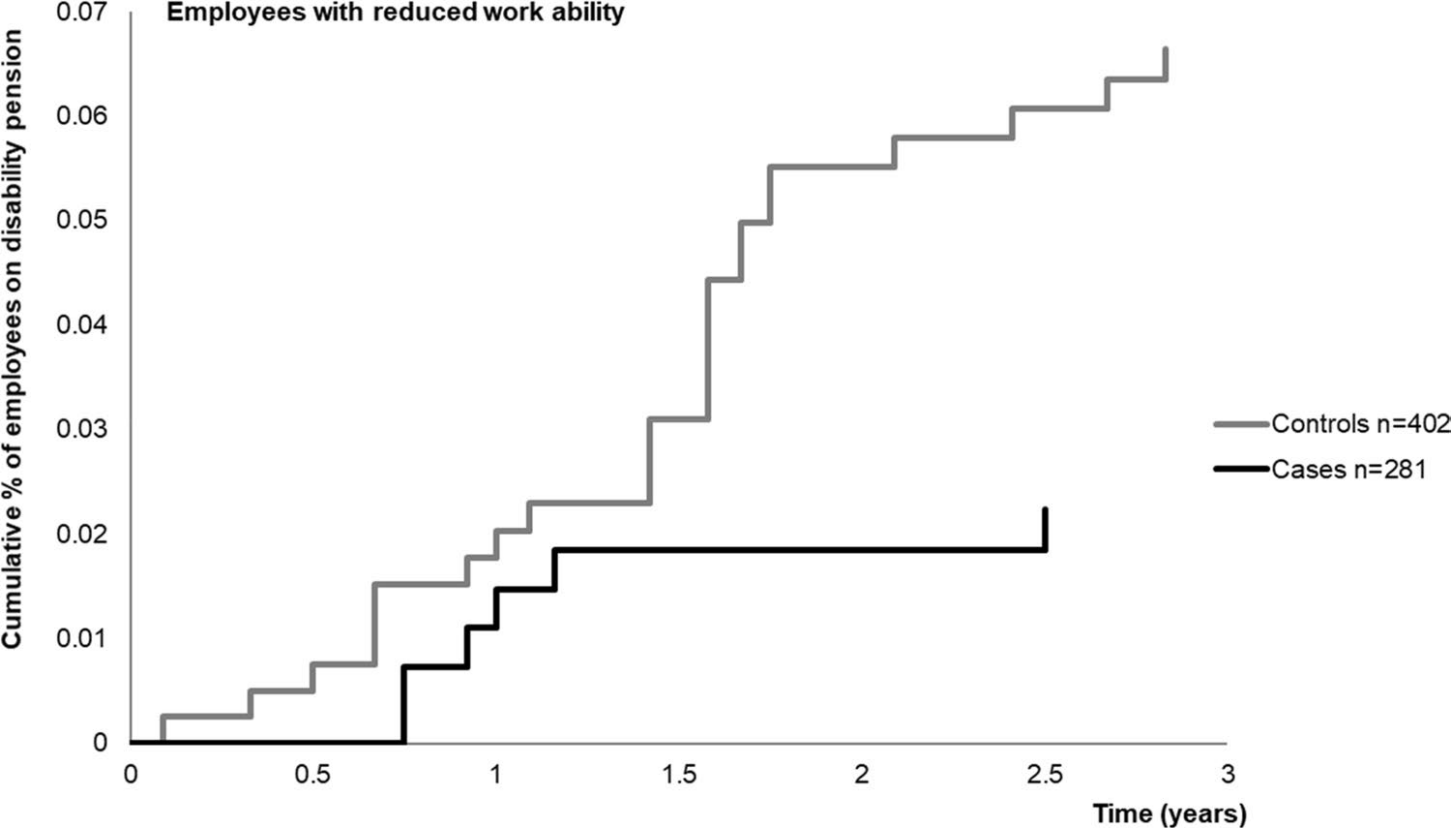
Cumulative incidence of disability retirement after implementation of RTW-coordinator model stratified by case/control status in employees with reduced work ability (Kaplan–Meier hazard functions)

In three years after implementing the RTW-coordinator model (post-intervention time), 28 (0.7%) employees among cases and 73 (1.3%) employees among controls were granted disability pension. In employees with reduced work ability, the corresponding figures were 6 (2.1%) and 25 (6.2%). After adjustment for sex, age, SES, job contract, psychiatric distress, previous long-term sickness absence (of > 30 days) and health behaviour (smoking, alcohol intake and physical inactivity), the risk of disability retirement was 2.0-fold among controls compared to cases (HR = 0.49, 95% CI 0.30–0.79) in the total population. In participants with reduced work ability, the association of the intervention with risk of disability retirement was greater as controls had approximately 2.9-fold risk of disability retirement compared to cases (HR = 0.34, 95% CI 0.12–0.99). (Table 3, Figs. 2, 3).

**Table 3:** Risk of disability retirement among cases (RTW-coordinator model implemented) (ref. controls, RTW-coordinator model not in use). Cox proportional hazards regression analysis

	Model 1			Model 2		
	Events/Total	HR	95% CI	Events/Total	HR	95% CI
Disability retirement, Total population (n = 9372)						
	97/9372	0.55	0.34–0.87	95/9226	0.49	0.30–0.79
Disability retirement, Reduced work ability (n = 658)						
	29/658	0.34	0.13–0.90	28/643	0.34	0.12–0.99

Model 1: Adjusted for sex, age, SES, and job contract

Model 2: Adjusted as Model 1 + psychiatric distress, previous sickness absence (of > 30 days) and health behaviour (smoking, alcohol intake and leisure-time physical inactivity)

## Discussion

Our findings suggest that the RTW-coordinator model may be associated with a lower risk of disability retirement among employees, but higher rates of sickness absence. We found that the risk of disability retirement (permanent exit from labour market) was reduced when the RTW-coordinator model was introduced in a municipality as compared to municipalities with no such model during the follow-up. The absolute difference in disability retirement incidence was relatively modest but not trivial as the cost of each disability retirement is substantial. The risk of sickness absence was increased when the RTW-coordinator model was introduced as compared to the situation when it was not applied. Among employees with reduced work ability, the difference between the groups was not statistically significant. Reduced work ability increases the risk of sickness absence, as was also seen from the differences in absolute number of sickness absence among those with reduced work ability compared to total population over a 7-year follow-up in our study. Keeping more people with reduced work ability in the labour market will plausibly increase sickness absence (temporary work disability) but may result in less permanent exits (permanent work disability) from the labour market.

A recent systematic review [10] reporting on 14 RCTs on RTW-coordination programmes found no benefits in terms of return-to-work outcomes. The authors called for more studies examining lasting return to work and applying a long follow-up time. Our study adds to existing literature with relatively long follow-up time as well as different measures of work disability. Outcome measures of both temporary and permanent work disability are important in the evaluation of potential benefits of RTW-programmes.

In addition to differences in outcomes, disparities in findings from different countries may reflect variation in the contexts [23] and implementation of the RTW-coordinator model [2]. In the Finnish municipal sector, RTW-coordinators are often placed within work organizations themselves and not in the health care sector, and thus they have a close workplace connection which has been recommended [3, 5, 9]. Work tasks of RTW-coordinators were nevertheless rather recently introduced in Finland and there is still large variation across employers and organizations and in their qualifications and tasks. When investigating the effectiveness of the RTW coordinator model, the type of programme used may possibly be a confounder. A need for formal guidelines or requirements for RTW-coordinators and a need to develop the RTW coordinator models further has been recognized [9].

The strength**s** of our study include its quasi-experimental study design (a real-life observational study setting as opposed to previous trials) and a large nationally representative data on the employees of municipal sector in Finland. Data on outcomes were register-based and the overall follow-up time was rather long. Selection to the intervention was not possible as in this quasi-experimental design all employees in the case group were exposed to the RTW-coordinator model and the intervention was not managed by researchers. Case and control municipalities were all large to medium-sized cities with rather similar socioeconomic structures and organizational contexts, even if there were some differences in the background characteristics of study participants in case and control groups. Pre-intervention trends of sickness absence were parallel in these groups. We applied difference-in -differences analysis to control for the fixed unobserved individual differences and common trends in case and control groups [20–22].

The limitations of this study include lack of information on the diagnoses of sick leaves and disability pensions. Future studies should for example differentiate between work disability due to musculoskeletal and mental disorders because the challenges in RTW after work disability due to musculoskeletal or mental disorders are different. RTW coordination may benefit these groups differently [9]. Also, musculoskeletal disorders other than rheumatoid arthritis were not included in illnesses under special reimbursement for medication, and therefore they were not covered by the definition of reduced work ability in this study. Given that musculoskeletal disorders are among the most common causes of sickness absence, this limitation may have contributed to the null finding on differences in sickness absence trends between cases and controls in the group of employees with reduced work ability.

Another limitation involves the generalizability of our findings. Lack of formal guidelines on RTW-coordinator model in Finland means the model is not well standardised limiting the generalizability of our findings as the implementation of RTW-coordinator model may vary between workplaces. However, there are some guidelines and recommendations suggesting that the implementation of RTW coordinator in public sector workplaces might be relatively similar across Finnish municipalities.

Reduced work ability increases the risk of sickness absence and, consistently with this notion, we observed higher rates of sickness absence among those with reduced work ability than the total population over a 7-year follow-up. It is therefore likely that keeping more people with reduced work ability in the labour market will increase sickness absence (temporary work disability) although also resulting in less permanent exits (permanent work disability) from the labour market. Further research carried out in different populations and settings (e.g. different social security systems with varying preconditions for the benefits) are needed to examine the extent to which the present findings are generalizable across different contexts. In addition, future cost–benefit analyses are also required to estimate the economic value of the policy.
